# Behavioral Consequences of a Combination of *Gad1* Haplodeficiency and Adolescent Exposure to an NMDA Receptor Antagonist in Long-Evans Rats

**DOI:** 10.3389/fphar.2021.646088

**Published:** 2021-03-30

**Authors:** Kazuyuki Fujihara, Takumi Sato, Kazuya Higeta, Yoshiki Miyasaka, Tomoji Mashimo, Yuchio Yanagawa

**Affiliations:** ^1^ Department of Genetic and Behavioral Neuroscience, Gunma University Graduate School of Medicine, Maebashi, Gunma, Japan; ^2^ Department of Psychiatry and Neuroscience, Gunma University Graduate School of Medicine, Maebashi, Gunma, Japan; ^3^ Institute of Experimental Animal Sciences, Graduate School of Medicine, Osaka University, Suita, Osaka, Japan; ^4^ Laboratory Animal Research Center, Institute of Medical Science, The University of Tokyo, Minato-ku, Tokyo, Japan

**Keywords:** γ-aminobutyric acid, GAD67, schizophrenia, MK-801 (dizocilpine), CRISPR/ Cas9

## Abstract

Glutamate decarboxylase 67-kDa isoform (GAD67), which is encoded by the *GAD1* gene, is one of the key enzymes that produce GABA. The reduced expression of GAD67 has been linked to the pathophysiology of schizophrenia. Additionally, the excitatory glutamatergic system plays an important role in the development of this disorder. Animal model studies have revealed that chronic blockade of NMDA-type glutamate receptors can cause GABAergic dysfunction and long-lasting behavioral abnormalities. Based on these findings, we speculated that *Gad1* haplodeficiency combined with chronic NMDA receptor blockade would lead to larger behavioral consequences relevant to schizophrenia in a rat model. In this study, we administered an NMDAR antagonist, MK-801 (0.2 mg/kg), to CRISPR/Cas9-generated *Gad1* ^+/−^ rats during adolescence to test this hypothesis. The MK-801 treated *Gad1* ^+/−^ rats showed a shorter duration in each rearing episode in the open field test than the saline-treated *Gad1* ^+/+^ rats. In contrast, immobility in the forced swim test was increased and fear extinction was impaired in *Gad1* ^+/−^ rats irrespective of MK-801 treatment. Interestingly, the time spent in the center region of the elevated plus-maze was significantly affected only in the saline-treated *Gad1* ^+/−^ rats. Additionally, the MK-801-induced impairment of the social novelty preference was not observed in *Gad1* ^+/−^ rats. These results suggest that the synergistic and additive effects of *Gad1* haplodeficiency and NMDA receptor blockade during adolescence on the pathogenesis of schizophrenia may be more limited than expected. Findings from this study also imply that these two factors mainly affect negative or affective symptoms, rather than positive symptoms.

## Introduction

γ-Aminobutyric acid (GABA) is a primary inhibitory neurotransmitter in the central nervous system (Obata, 2013). Post-mortem brain studies on schizophrenia have shown that GABAergic disturbances are part of the pathophysiology of the disorder (Lewis and Sweet, 2009). In particular, the expression level of the GABA-synthesizing enzyme glutamate decarboxylase 67-kDa isoform (GAD67) is lower in the cerebral cortex of patients with schizophrenia than in that of healthy subjects (Guidotti et al., 2000; Volk et al., 2000; Hashimoto et al., 2003; Hashimoto et al., 2008; Curley et al., 2011). GAD67 is encoded by the *GAD1* gene, whose SNPs are also suggested to be risk factors for schizophrenia (Addington et al., 2005). We previously reported that *Gad1* ^−/−^ rats displayed some schizophrenia-relevant behaviors, including working memory, which is important for the functional outcome of schizophrenia (Fujihara et al., 2020a). However, a major difference between the model animals and humans with schizophrenia is the complete loss of GAD67 in *Gad1* ^−/−^ rats. Therefore, the behavioral consequences of a more moderate reduction of GAD67, such as in *Gad1* ^+/−^ rats, should be assessed. Considering that *Gad1* ^+/−^ mice are more sensitive to maternal and fetal stresses than wild-type mice (Uchida et al., 2011) and show a reduction in the number of parvalbumin (PV)-positive GABAergic neurons, as seen in schizophrenia (Uchida et al., 2014), *Gad1* haplodeficient rodents may be useful as model animals for testing vulnerability to schizophrenia.

The glutamatergic system, the most abundant excitatory neuron in the cerebrum, is also implicated in the pathogenesis of schizophrenia (Krystal et al., 1994). For instance, the administration of phencyclidine, an antagonist of the NMDA-type glutamate receptor, induces a wide range of schizophrenic symptoms in humans and rodent models (Lee and Zhou, 2019). Other NMDA receptor antagonists, such as ketamine and MK-801, have similar effects and are frequently used to establish a pharmacological model of schizophrenia (Kawabe et al., 2007; Kawabe, 2017; Unal et al., 2018; Pérez et al., 2019). Since the most susceptible age for the disorder is from adolescence to young adulthood, chronic pharmacological blockade of NMDA during this period is a possible model for schizophrenia. Such animal models show impaired spatial cognition (Rujescu et al., 2006; Li et al., 2016), enhanced anxiety-like behaviors (Uttl et al., 2018; Pérez et al., 2019), decreased social novelty preference (Pérez et al., 2019), and impaired prepulse inhibition (Ma et al., 2020). The molecular details underpinning these behavioral alterations are still unclear. Some studies have shown that repetitive administration of NMDA receptor antagonists induces a reduction in the number of PV-positive GABAergic neurons (Rujescu et al., 2006; Li et al., 2016) and decreases the expression level of vesicular GABA transporter, which plays an important role in the vesicular packing and release of GABA (Ma et al., 2020). Hence, these results suggest that chronic dysfunction of NMDA receptors also leads to GABAergic disruptions.

Therefore, we hypothesized that the combination of genetic predisposition in the GABAergic system and the pharmacological blockade of NMDA receptors can cause a more severe schizophrenia-like behavioral phenotype, including working memory impairment in rats. In this preliminary study, we used *Gad1* ^+/−^ rats as model animals to test this hypothesis. At present, the CRISPR/Cas9 technique has become a powerful tool for generating knockout animals, including mice and other species (Cong et al., 2013; Leo et al., 2018). In studies on psychiatric disorders, using rats rather than mice has certain advantages. For example, performing a surgical operation for *in vivo* recording is easier and conducting more complicated behavioral tasks is more feasible. The current study may also provide a basic behavioral characterization of the model for further studies that may wish to exploit these advantages.

## Materials and Methods

### Animals

We generated *Gad1* knockout Long-Evans rats using the CRISPR/Cas9 system. The detailed procedure for the generation and validation of *Gad1* knockout rats has been reported elsewhere (Fujihara et al., 2020a). To obtain the rats for the present study, we crossed male and female *Gad1* ^+/−^ (heterozygous *Gad1* knockout, HET) rats. All experiments in this study were approved by the Animal Care and Experimentation Committees of Gunma University (Permission number: 19-009) and the Animal Research Committee of Osaka University (Permission number: 24-006-042). Food (CLEA Rodent Diet CE-2, Clea Japan, Meguro, Tokyo, Japan) and water were provided *ad libitum* (Fujihara et al., 2020a). Every effort was made to minimize the number of animals used and reduce their suffering.

### MK-801 Administration

From postnatal days 30–43, we intraperitoneally administered either (+)-MK-801 (Sigma-Aldrich, St. Louis, MO, United States), an NMDA receptor antagonist, or saline once a day during the light phase (Uttl et al., 2018; Ma et al., 2020). The reported dosage of MK-801 varied in the literature. In this study, we used 0.2 mg/kg (Ma et al., 2020) to test the combined effect of moderate GAD67 reduction and moderate NMDA receptor blockade. Each rat of each genotype (*Gad1* ^+/+^ or *Gad1* ^+/−^) was assigned to one of the two conditions (saline or MK-801) using a simple randomization method. The experimental unit was an individual animal, not a cage. Four groups were prepared: 1) MK-801 treated *Gad1* ^+/−^ (HET-MK); 2) saline-treated *Gad1* ^+/−^ (HET-SAL); 3) MK-801 treated *Gad1* ^+/+^ (WT-MK); 4) and saline-treated *Gad1* ^+/+^ (WT-SAL). The body weight of the rats was measured during the 14-days administration of MK-801. We observed no differences in the growth curves among the groups (see Supplementary Figure S1).

### Behavioral Analysis

Each rat was given an individually identifiable ear punch, and the experimenters were blinded to the genotype and drug treatment of each rat. Seven days after the last injection of MK-801, we started the behavioral test battery. The acoustic startle response (including a prepulse inhibition [PPI]), Y-maze, elevated plus-maze, open field, social interaction, forced swim, and fear conditioning tests were conducted as described previously with minor modifications (Fujihara et al., 2015a; Miyata et al., 2019; Fujihara et al., 2020a, Fujihara et al., 2020b). The test battery was performed in the above-mentioned order (see Supplementary Table S1). The detailed procedures of the behavioral tests are described in the Supplementary Material section (see Supplementary Methods). The interval between each test was more than 5 days to curtail the stress of the rats. All apparatuses and the software for behavioral analysis were supplied by a single vendor (O'Hara & Co., Ltd., Tokyo, Japan).

### Data Analysis

Group comparisons were carried out using a two-way analysis of variance (ANOVA) or three-way repeated-measures ANOVA. Intragroup comparisons were analyzed using *t-*tests. If interactions such as genotype × drug, were significant, we performed *post hoc* simple main effect tests (Bonferroni-adjusted). Additionally, if significant or subthreshold effects were observed in both the genotype and the drug, we applied Dunnett’s test to assess the additive effect. The WT-SAL group was used as the control for Dunnett’s test. *p*-values < 0.05 were considered significant. As the present study was exploratory, we reported subthreshold level *p*-values (i.e., 0.05 ≤ *p* ≤ 0.1) with corresponding effect sizes. Partial *η*-squared (*η*_p_^2^) and Cohen’s *d* values were calculated to assess the effect sizes. Sphericities were assessed using Mauchly's test in the case of a three-way repeated-measures ANOVA. If the null hypothesis of Mauchly’s test was rejected, a Greenhouse-Geisser correction was applied. Statistical analyses were performed using the statistical software SPSS (SPSS Inc., Chicago, IL, United States) and R (https://www.r-project.org/) (R Core Team, 2017). Graphs were generated using GraphPad Prism (GraphPad Software, Inc., La Jolla, CA, United States). Only the key statistical results are described in the main text. Please refer to the Supplementary Materials section for more detail.

## Result

### HET-MK Rats Displayed Shorter Rearing Durations in the Open Field Test

**FIGURE 1:**
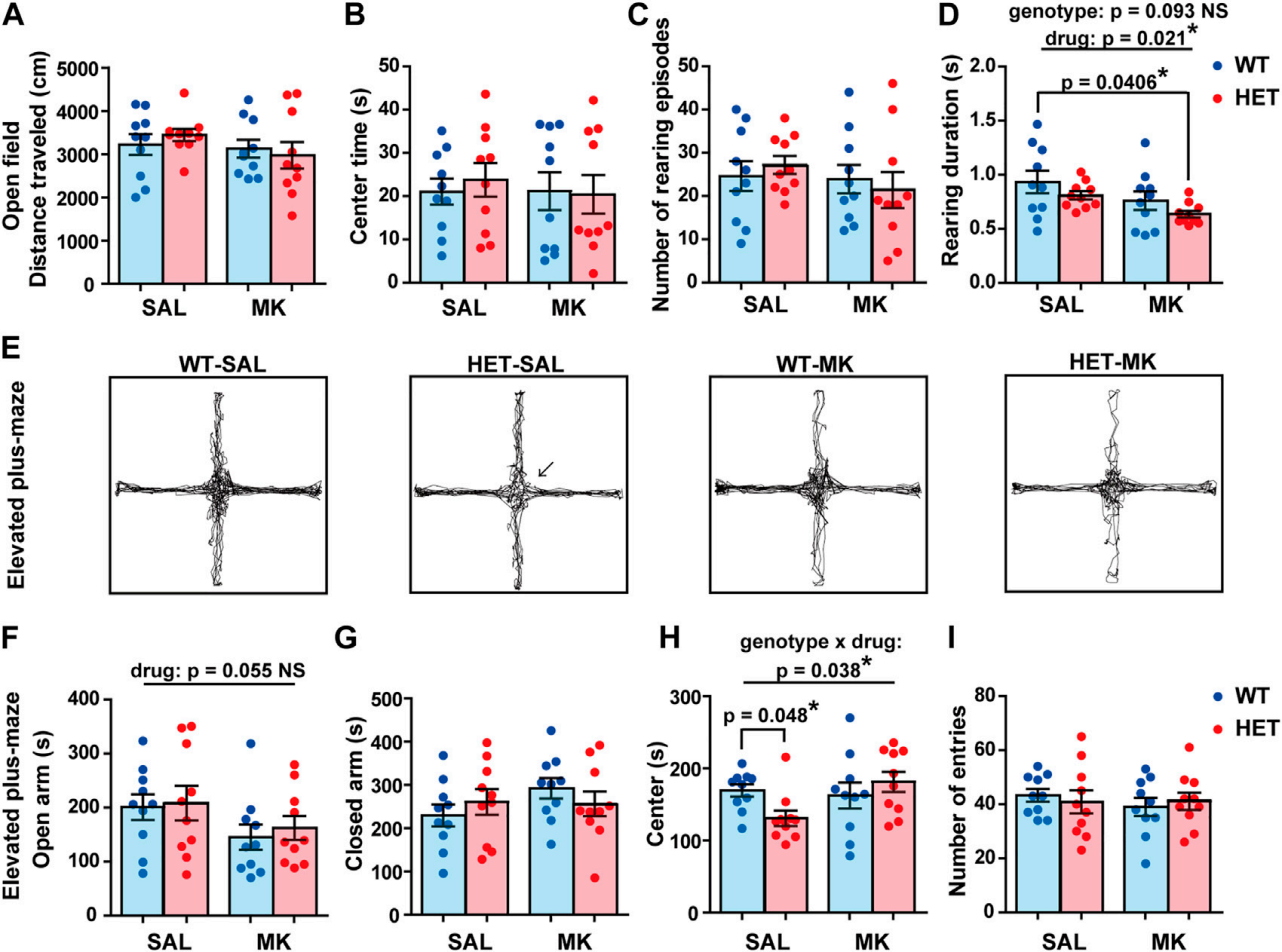
Exploratory and anxiety-like behaviors were influenced by *Gad1* genotype and MK-801 treatment. In the open field test, **(A)** the distance traveled, **(B)** exploration time of the center region, and the number of rearing episodes were comparable between the four groups. The rearing duration for each episode was significantly reduced by MK-801 treatment (*p* = 0.021). The effect of genotype on the rearing duration was a subthreshold level (*p* = 0.093). However, as a result of the combination of these two effects, the HET-MK group showed a significantly shorter duration than that of the WT-SAL (Dunnett’s test; *p* = 0.0406). **(E)** Representative trajectories in the elevated plus-maze test. The vertical arms were open while the horizontal arms were closed respectively. HET-SAL groups stayed less time in the center area of the maze (arrow). **(F)** The time spent on the open arms was not significantly different among the four groups, but a subthreshold level effect of MK-801 was observed (*p* = 0.055). **(G)** The time spent on the closed arm was not significantly different between the groups. **(H)** A significant effect of genotype × drug interaction was observed in the time spent in the center area. The center time was reduced in HET rats only in the SAL-treated condition. **(I)** The number of entries to the arms was similar in the four groups. The results are presented as average ±SEM. Data were analyzed using two-way ANOVA. If a significant interaction of gene × drug was observed, a *post hoc* simple main effect test was performed (Bonferroni adjusted). If significant or subthreshold effects were observed both in genotype and drug, a Dunnett’s test was carried out to assess the additive effect. WT: wild-type, HET: *Gad1* ^+/−^, SAL: saline, MK: MK-801. *p < 0.05, **p < 0.01; NS, not significant.

In the open field test, comparable distances were traveled by all the groups (Figure 1A) and center times were similar (Figure 1B). Although the number of rearing episodes was not significantly different between the groups (Figure 1C), the duration of each rearing was significantly reduced by the MK-801 treatment (Figure 1D; drug, *F* (1,36) = 5.863, *p* = 0.021, *η*_p_^2^ = 0.140). A subthreshold level effect of genotype was also observed with a medium effect size (genotype, *F* (1,36) = 2.986, *p* = 0.093, *η*_p_^2^ = 0.077). As an additive effect of drug and genotype, the HET-MK rats had a significantly shorter rearing duration than the WT-SAL rats (Dunnett’s test, corrected *p* = 0.0406). The results implied that these factors altered exploratory behavior slightly, but significantly in a novel environment.

### Complex Effects of *Gad1* Haplodeficiency and MK-801 Administration on Anxiety-like Behaviors

We assessed anxiety-like behaviors using the elevated plus-maze test (Figures 1E–I). MK-801 administration resulted in a decrease in the time spent in the open arms with a medium effect size irrespective of the genotype. However, the effect was under the statistically significant threshold (Figure 1F; drug, *F* (1,36) = 3.934, *p* = 0.055, *η*_p_^2^ = 0.099; genotype, *F* (1,36) = 0.229, *p* = 0.635, *η*_p_^2^ = 0.006; genotype × drug, *F* (1,36) = 0.035, *p* = 0.853, *η*_p_^2^ = 0.001). The time spent in the closed arms did not differ among the four groups (Figure 1G). However, we observed a significant genotype × drug interaction in the time spent in the center region (Figure 1H; genotype, *F* (1,36) = 0.535, *p* = 0.469, *η*_p_^2^ = 0.015; drug, *F* (1,36) = 2.693, *p* = 0.110, *η*_p_^2^ = 0.070; genotype × drug, *F* (1,36) = 4.665, *p* = 0.038, *η*_p_^2^ = 0.115). Contrary to our expectations, a significant difference between the two genotypes was found only in the SAL condition: the HET-SAL rats spent less time in the center region than the WT-MK rats (Figure 1E, indicated by an arrow; simple main effect, corrected *p* = 0.048). Finally, the distance traveled in the elevated plus-maze test was comparable in all groups (Figure 1I). These results suggested that these two factors differentially affected behaviors in the elevated plus-maze test.

### *Gad1* Haplodeficiency Prevented MK-801-Induced Impairment of Social Novelty Preference

**FIGURE 2:**
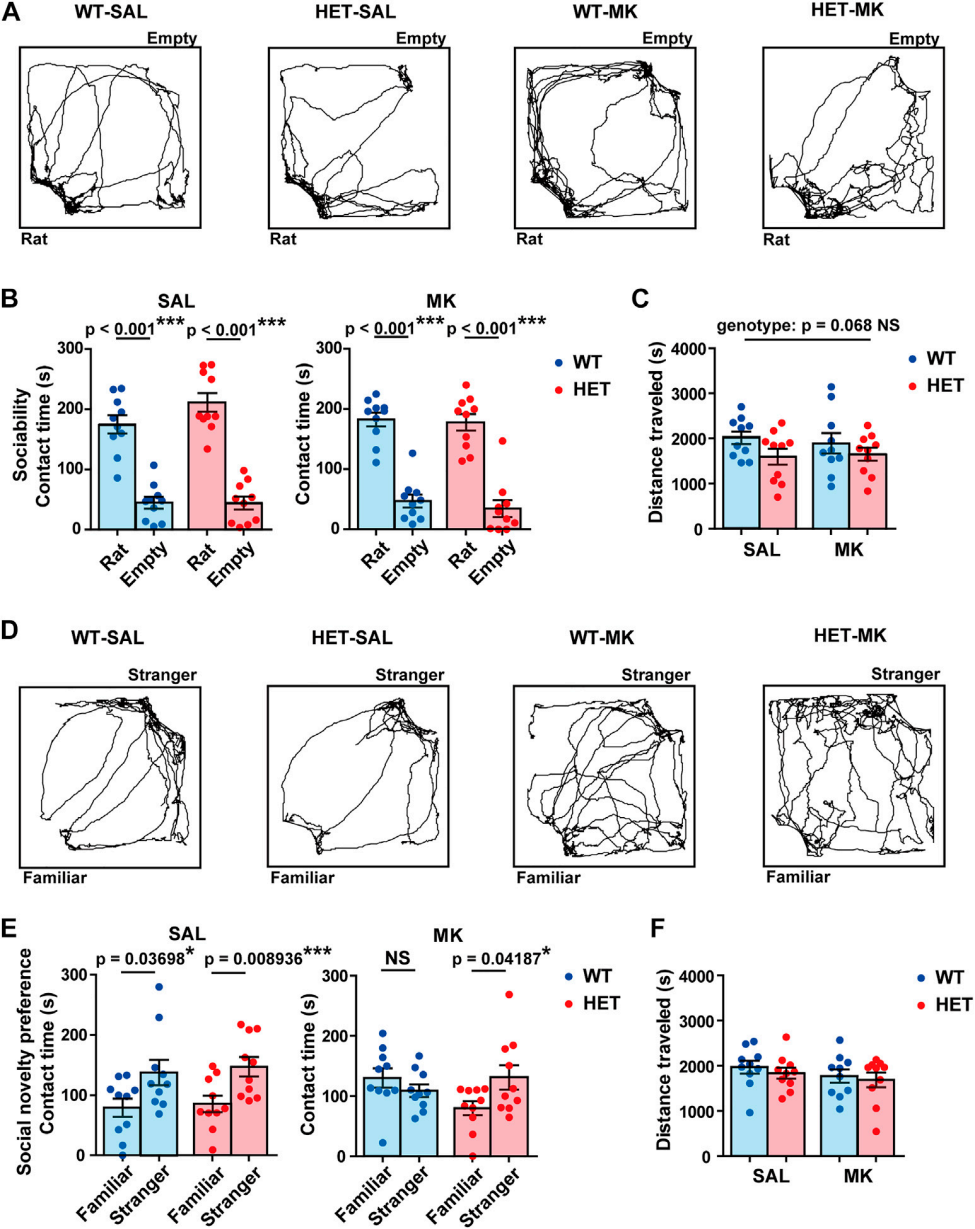
MK-801 treatment affected the social novelty preference only in *Gad1* WT rats. **(A)** Representative trajectories of rats in the sociability test. **(B)** All the groups preferred the rat-enclosed cage to the empty cage. **(C)** Although the distance traveled during the sociability test was not significantly different between the groups, a subthreshold effect of genotype was found (*p* = 0.068). **(D)** Representative trajectories of rats in the social novelty preference test. **(E)** SAL-treated rats of both genotypes spent a longer time in contact with a stranger rat than they did with a familiar rat. MK-801 treatment diminished this trend in WT rats but not in HET rats. **(F)** The distance traveled during the social novelty preference test was similar between the four groups. The results are presented as average ±SEM. Data were analyzed using a *t*-test **(B,C)** or two-way ANOVA **(C,F)**. If a significant interaction of gene × drug was observed, a *post hoc* simple main effect test was performed. WT: wild-type, HET: *Gad1* ^+/−^, SAL: saline, MK: MK-801. *p < 0.05, **p < 0.01, ***p < 0.01; NS, not significant.

In the sociability test, all four groups stayed for a longer time around the cage with a stranger rat than they did around the empty cage (Figures 2A,B; WT-SAL, *t* (18) = 7.2352, *p* < 0.001, Cohen’s *d* = 3.2357; HET-SAL, *t* (18) = 8.8649, *p* < 0.001, Cohen’s *d* = 3.945; WT-MK, *t* (18) = 8.5758, *p* < 0.001, Cohen’s *d* = 3.8352; HET-MK, *t* (18) = 7.2807, *p* < 0.001, Cohen’s *d* = 3.2561). The distance traveled in the HET group was slightly reduced compared with that in the WT group with a medium effect size, but the genotype effect was not statistically significant (Figure 2C, genotype, *F* (1,36) = 3.536, *p* = 0.068, *η*_p_^2^ = 0.089; drug, *F* (1,36) = 0.039, *p* = 0.844, *η*_p_^2^ = 0.010; genotype × drug, *F* (1,36) = 0.257, *p* = 0.615, *η*_p_^2^ = 0.070).

In the subsequent social novelty preference test, the WT-SAL, HET-SAL, and HET-MK groups spent more time in contact with stranger rats than they did with familiar rats (Figures 2D,E; WT-SAL, *t* (18) = 2.2528, *p* = 0.037, Cohen’s *d* = 1.0075; HET-SAL, *t* (18) = 2.9305, *p* = 0.0089, Cohen’s *d* = 1.3106; HET-MK, *t* (18) = 2.1907, *p* = 0.0419, Cohen’s *d* = 0.9797), but this trend was not observed in the WT-MK group (*t* (18) = 1.1164, *p* = 0.2789, Cohen’s *d* = 0.4993). This result suggested that *Gad1* haplodeficiency may have a preventive effect against MK-801-induced abnormalities in social behavior. Furthermore, the distance traveled was similar in all the groups (Figure 2F; genotype, *F* (1,36) = 0.558, *p* = 0.460, *η*_p_^2^ = 0.015; drug, *F* (1,36) = 1.453, *p* = 0.236, *η*_p_^2^ = 0.039; genotype × drug, *F* (1,36) = 0.028, *p* = 0.868, *η*_p_^2^ = 0.001).

### Neither *Gad1* Haploinsufficiency nor MK-801 Treatment Affected Working Memory

Impaired working memory is a cognitive symptom in patients with schizophrenia (Keefe and Harvey, 2012). To screen spatial working memory in our model rats, we measured the spontaneous alternation behavior in the Y-maze using a previously reported method (Fujihara et al., 2015a; Fujihara et al., 2020a). The alternation rate in each group was higher than 50%. This was a chance-level performance rate of the task. There were no significant main effects between these factors in genotype, drug, or interaction (Supplementary Figure S2). Findings from this study confirmed that working memory was not affected by these two factors.

### *Gad1* Haploinsufficiency Reduced Acoustic Startle Amplitude Without Affecting PPI

**FIGURE 3:**
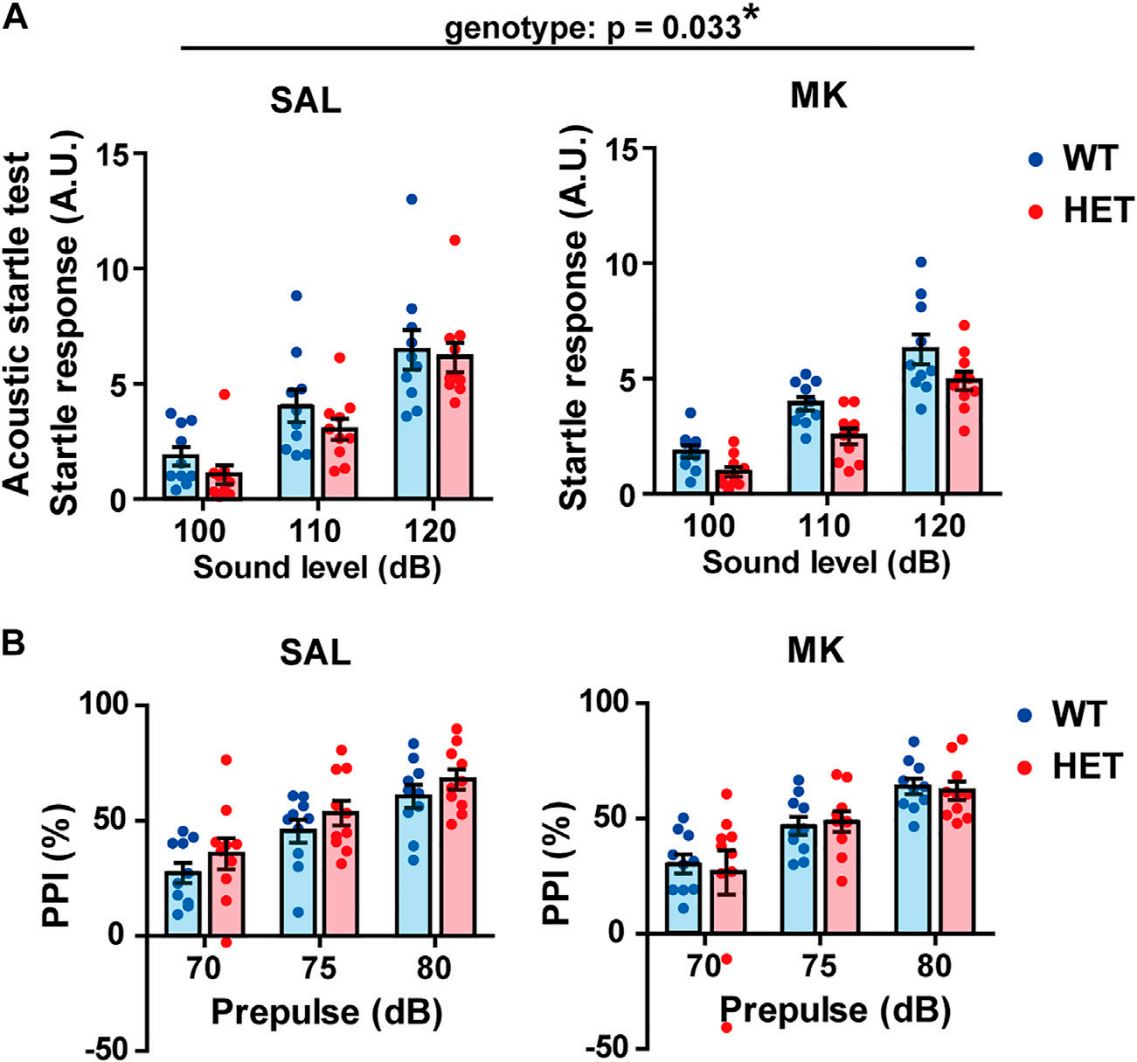
Acoustic startle response was decreased in *Gad1* HET rats. **(A)** Startle amplitudes of HET rats were lower than those of WT rats irrespective of MK-801 treatment (*p* = 0.033). **(B)** Prepulse inhibition (PPI) was not affected by *Gad1* genotype and MK-801 treatment. The results are presented as average ± SEM. Data were analyzed using three-way repeated-measures ANOVA. If a significant interaction of gene × drug was observed, a *post hoc* simple main effect test was performed. WT: wild-type, HET: *Gad1* ^+/−^, SAL: saline, MK: MK-801. *p < 0.05.

Reduced PPI in the acoustic startle response is a hallmark of schizophrenia-like behavior in animal models (Fujihara et al., 2015a). First, we performed an acoustic startle test at three different intensities of sound (100, 110, and 120 dB) (Figure 3A). The startle amplitudes significantly increased in a dose-dependent manner with the sound intensities (sound intensity, *F* (1.691, 60.875) = 202.499, *p* < 0.001, *η*_p_^2^ = 0.849). No significant genotype × drug × sound intensity interaction or genotype × drug interaction was detected (genotype × drug × sound, *F* (1.691, 60.875) = 0.578, *p* = 0.536, *η*_p_^2^ = 0.016; genotype × drug, *F* (1, 36) = 1.910, *p* = 0.336, *η*_p_^2^ = 0.009). However, the HET rats showed lower startle amplitudes with a relatively large effect size (genotype, *F* (1, 36) = 4.931, *p* = 0.033, *η*_p_^2^ = 0.120). Thus, MK-801 treatment did not influence startle responses (drug, *F* (1, 36) = 0.707, *p* = 0.406, *η*_p_^2^ = 0.019).

Next, we tested possible impairments in PPI (Figure 3B). Among the groups, the higher the prepulse intensity, the greater the PPI (prepulse, *F* (1.366, 49.184) = 140.961, *p* < 0.001, *η*_p_^2^ = 0.797). There was no genotype × drug × prepulse interaction (*F* (1.366, 49.184) = 0.298, *p* = 0.659, *η*_p_^2^ = 0.008). Although the HET-SAL and HET-MK rats showed reduced startle amplitude, their PPI was similar to that of the WT rats (genotype, *F* (1, 36) = 0.487, *p* = 0.490, *η*_p_^2^ = 0.013). There was no significant drug effect or genotype × drug interaction (drug, *F* (1, 36) = 0.172, *p* = 0.681, *η*_p_^2^ = 0.005; genotype × drug, *F* (1, 36) = 0.916, *p* = 0.345, *η*_p_^2^ = 0.025).

### *Gad1* Haploinsufficiency Resulted in Increased Immobility in the Forced Swim Test

**FIGURE 4:**
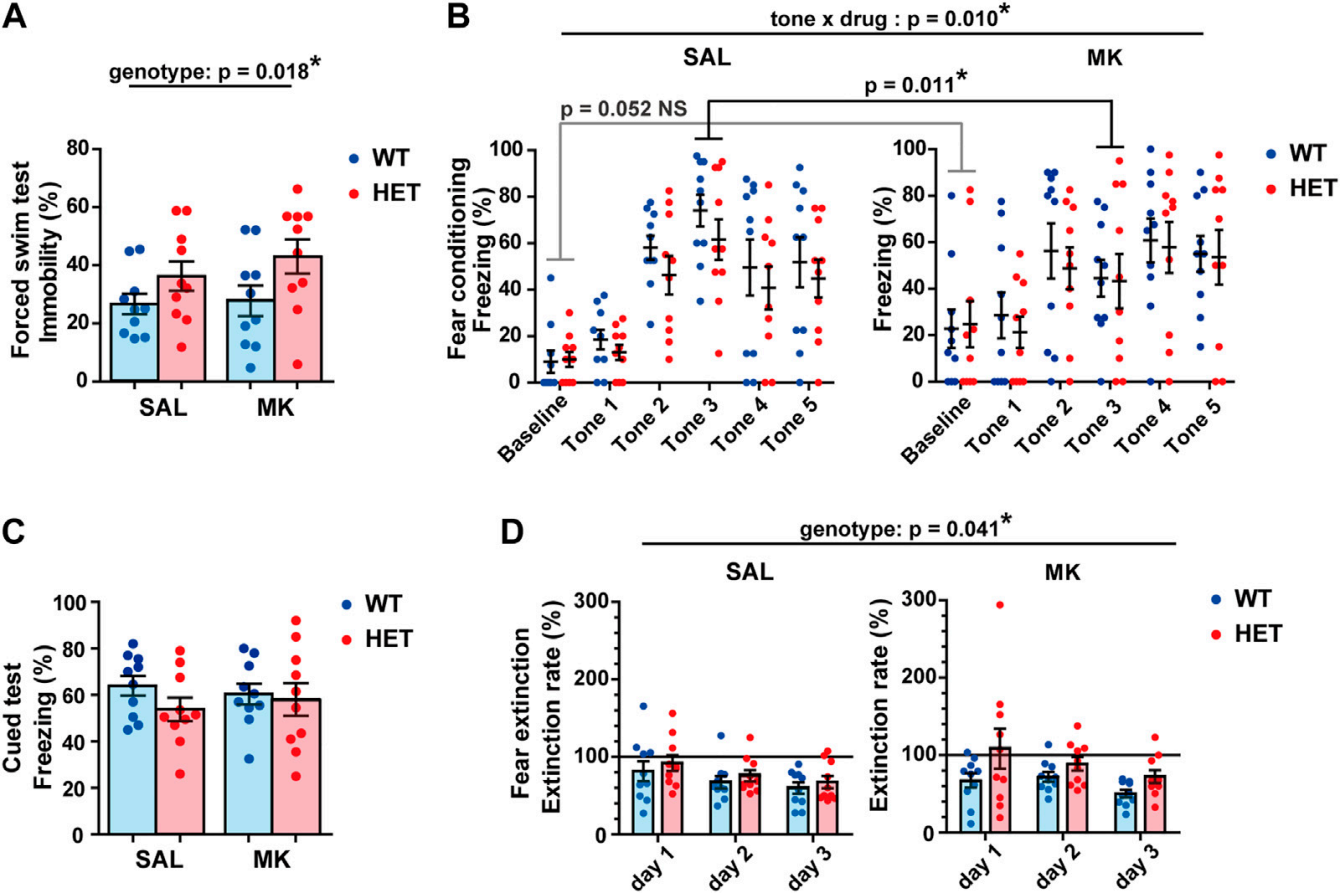
*Gad1* HET rats showed increased immobility and delayed fear extinction in the forced swim test. **(A)** HET rats showed a longer immobility duration during the forced swim test (*p* = 0.018), while MK-801 treatment did not have any effect. **(B)** The training for cued fear conditioning. MK-801 treatment altered the time course of the freezing level during training (tone × drug, *p* = 0.010). The baseline freezing was slightly higher in MK groups but it was at a subthreshold level (*p* = 0.052). However, the freezing time of MK groups was significantly shorter in tone 3 (*p* = 0.011). **(C)** The freezing time during the cued test was comparable between the groups. **(D)** HET rats displayed a slight but significant delay in fear extinction irrespective of MK-801 treatment (*p* = 0.041). The results are presented as average ±SEM. Data were analyzed using a two-way ANOVA **(A,C)** or three-way repeated-measures ANOVA **(B,D)**. If a significant interaction between two or three factors was observed, a *post hoc* simple main effect test was performed (Bonferroni adjustment). WT: wild-type, HET: *Gad1* ^+/−^, SAL: saline, MK: MK-801. *p < 0.05; NS, not significant.

We tested depression-like or negative symptom-like behaviors using the forced swim test. During the test, the HET rats showed increased immobility throughout the session, irrespective of MK-801 treatment (Figure 4A). We found a significant main effect related to the genotype. However, no significant main effect or significant interaction of genotype × drug was observed (genotype main effect, *F* (1,36) = 6.1750, *p* = 0.0180, *η*_p_^2^ = 0.1460; drug main effect, *F* (1,36) = 0.6120, *p* = 0.4390, *η*_p_^2^ = 0.0170; genotype × drug interaction, *F* (1,36) = 0.3190, *p* = 0.5760, *η*_p_^2^ = 0.009).

### *Gad1* Haploinsufficiency Delayed the Extinction of Cued Fear Memory

Fear expression and fear memory were assessed using a cued fear conditioning test. In the training phase, the four groups showed comparable freezing durations overall (Figure 4B). Although the freezing duration increased with tone-shock pairings (tone, *F* (3.412, 122.843) = 23.209, *p* < 0.001, *η*_p_^2^ = 0.392), the time course of freezing was affected by MK-801 treatment (drug × tone, *F* (3.412, 122.843) = 3.7210, *p* = 0.010, *η*_p_^2^ = 0.0940). In particular, the baseline freezing was slightly higher in the MK groups, but it was at a subthreshold level (simple main effect, corrected *p* = 0.052). Additionally, the MK groups showed significantly lower freezing during tone 3 (simple main effect, corrected *p* = 0.011).

On the second day of the experiment, rats were placed in a box with a different context and only the tones were administered. There was no difference in the freezing levels between the four groups (Figure 4C). We then repeated the same cued test every day to assess for fear extinction (Figure 4D). The decrease in freezing level in each rat was expressed as a ratio to the freezing level from the first cued test (extinction rate [%]). In the extinction sessions, freezing levels of all groups decreased over the course of 3 days (day, *F* (1.367, 49.202) = 8.020, *p* = 0.003, *η*_p_^2^ = 0.182). However, the HET rats showed significantly slower decrease in freezing than did the WT rats (genotype, *F* (1, 36) = 4.486, *p* = 0.041, *η*_p_^2^ = 0.111). There were no significant main effects of the drug, genotype × drug interaction, or the genotype × drug × day interaction (drug, *F* (1, 36) = 0.089, *p* = 0.767, *η*_p_^2^ = 0.002; genotype × drug, *F* (1, 36) = 1.119, *p* = 0.297, *η*_p_^2^ = 0.030; genotype × drug × day, *F* (1.367, 49.202) = 0.433, *p* = 0.575, *η*_p_^2^ = 0.012).

## Discussion

In the present study, we examined the possible interactions or additive effects of *Gad1* haplodeficiency and chronic blockade of NMDA receptors in rats during adolescence. We found that such effects were limited to rearing in the open field test, center time in the elevated plus-maze test, and social novelty preference in the social interaction test. It should be noted that the effect sizes of the alterations in this study were not large, and the directions of the changes were contrary to our expectations. Additionally, we found no effects on performance in the Y-maze test. Therefore, our hypothesis that these two factors cause impairment of working memory was not supported by the study findings. In our previous study, *Gad1* ^−/−^ rats showed lower performance in the Y-maze and the eight-arm radial-maze tests than *Gad1* ^+/+^ rats (Fujihara et al., 2020a). Since the sensitivity of the Y-maze test in detecting working memory impairment was relatively low, as we pointed out previously (Fujihara et al., 2020a), more complex tasks, such as the eight-arm radial-maze test should be further analyzed in the future. In any case, the combination of *Gad1* haplodeficiency and chronic blockade of NMDA receptors was not sufficient to induce cognitive impairment comparable to that of *Gad1* ^−/−^ rats.

Neonatal administration of MK-801 and homozygous elimination of *Gad1* decreases the frequency of rearing in the open field test in rats (Kawabe et al., 2007; Fujihara et al., 2020a). In the present study, although the MK-801 treatment during adolescence did not reduce the number of rearing events, it did slightly reduce the duration of each rearing event. We also found that *Gad1* haplodeficiency resulted in a trend-level reduction in rearing duration. As a result, the combination of MK-801 and *Gad1* haplodeficiency caused a significant reduction in rearing duration in the HET-MK group, although the reduction was weaker compared to that in *Gad1* ^−/−^ rats. The mechanism by which rearing is reduced remains elusive; however, these reduced exploratory behaviors may be associated with negative symptoms of schizophrenia, as suggested in our previous study (Fujihara et al., 2020a).

According to brain imaging studies in human subjects, the GABAergic system is involved in the control of impulsive behavior (Silveri et al., 2013; Fujihara et al., 2015b). We previously reported that the lower the GABA concentrations in the anterior cingulate gyrus, the shorter the time for decision-making, leading to more impulsive behaviors (Fujihara et al., 2015b). Although the interpretation of the center time in the elevated plus-maze test is controversial, some researchers consider it to reflect decision-making (Rodgers and Dalvi, 1997; Albani et al., 2015). Based on this interpretation, the reduced center time in the HET-SAL rats is consistent with the findings of a previous human study (Fujihara et al., 2015b). Whether GABAergic neurotransmission in the anterior cingulate gyrus is involved in this phenotype of the HET-SAL rats should be clarified in the future, using a region-specific knockout or knockdown experiment. Interestingly, in the MK-801-treated condition, the center time was comparable between the HET and WT rats. However, we should not simply conclude that this has a therapeutic effect. In the present study, MK-801 treatment also reduced the time spent in the open arms, although the effect was at a subthreshold level (*p* = 0.055). This result suggests that chronic MK-801 treatment during adolescence may alter anxiety levels, as reported previously (Uttl et al., 2018; Pérez et al., 2019). Consequently, the decision-making process may be affected by the altered anxiety level. In future studies, the impulsivity in the HET-SAL and HET-MK groups should be addressed in a more specialized way (e.g., Go/No-Go and five-choice serial reaction time tasks).

We found significant main effects of *Gad1* haplodeficiency in several behavioral tests, irrespective of MK-801 treatment. First, the HET rats unexpectedly displayed attenuated acoustic startle responses. The reduced startle amplitude may reflect possible impairment in auditory function, motor function, or emotional processing (Umemori et al., 2013). However, the PPI and cued fear memory were normal in the HET rats, suggesting that auditory function itself was not significantly affected. However, a behavioral study on *Gad1* ^+/−^ mice did not show such a significant reduction in the acoustic startle response (Nullmeier et al., 2020). This discrepancy may be attributed to differences in the species and variations in experimental conditions. Given that *Gad1* ^−/−^ mice are associated with 100% lethality and *Gad1* ^−/−^ rats can survive to adulthood (Asada et al., 1997; Fujihara et al., 2020a), it is conceivable that differences in other phenotypes may be found. Second, as reported recently in *Gad1* ^+/−^ mice (Nullmeier et al., 2020), the immobility time in the forced swim test was also prolonged in the HET rats in the present study. Enhanced immobility is considered to be a negative symptom-like or depressive-like behavior in rodent models (Noda et al., 1995; Nullmeier et al., 2020). Curiously, *Gad1* ^−/−^ rats show less immobility (rather than hyperactivity) in the forced swim test (Fujihara et al., 2020a). Therefore, the relationship between the number of intact alleles of *Gad1* and immobility is nonlinear. Third, we also observed a slight delay in fear extinction in the HET group. This result is similar to the findings of previous studies in amygdala-specific *Gad1* knockdown mice (Heldt et al., 2012), PV neuron-specific *Gad1* knockdown mice (Brown et al., 2015), and *Gad1* ^−/−^ rats (Fujihara et al., 2020b). Considering that amygdala-specific knockdown is sufficient to cause this phenotype, the reduction of GAD67 and subsequent impairment in GABAergic transmission in the amygdala may be responsible for this behavior in HET rats as well.

The most bizarre phenomenon observed in the present study was the absence of the MK-801-induced reduction in social novelty preference in HET rats. The blockade of NMDA receptors during adolescence impairs social novelty preference in mice (Pérez et al., 2019). Such changes in social behavior are thought to be associated with reductions in GAD67 and PV neurons in the prefrontal cortex (Pérez et al., 2019). The reason why the HET rats were resistant to the effect of MK-801 in terms of social novelty preference can be explained as follows: the secondary changes caused by congenital *Gad1* haplodeficiency showed a defensive effect against MK-801. For example, another GAD, GAD65 (encoded by *Gad2*), was compensatorily upregulated in *Gad1* ^−/−^ rats. Although not significant, a trend-level increase in GAD65 was also observed in the HET rats in our previous study (Fujihara et al., 2020a). It is unlikely that GAD65 compensated for all the GAD67 functions because we found some significant effects of *Gad1* haplodeficiency in the present study. Future studies should explore the secondary changes caused by *Gad1* haplodeficiency and their possible effects on social behaviors in future studies.

In summary, our study is the first report on the behavioral consequences of the combination of classical pharmacological blockade and genetic predisposition to the GABAergic system. However, in terms of the pathophysiology of schizophrenia, neither *Gad1* haplodeficiency, or NMDA receptor blockade, nor the combination of the two, reproduced all of the symptoms relevant to schizophrenia. Additionally, we revealed that the behavioral alterations were mainly related to negative symptoms, decision-making, and emotional domain, rather than positive symptoms and working memory impairment. These findings provide a foundation for future studies on the pathogenesis of schizophrenia. Current treatment strategies for schizophrenia are still unsatisfactory. Atypical antipsychotics have limited therapeutic efficacy for negative symptoms (Remington et al., 2016). In addition to the positive symptoms, new treatment strategies are needed for managing the negative symptoms. Recently, new therapeutic strategies, including drugs that enhance NMDA receptor neurotransmissions, such as sodium benzoate (Lane et al., 2013; Lin et al., 2017; Lin et al., 2018) and sarcosine (Chang et al., 2020), and drugs that enhance GABA neurotransmissions, such as α5 GABAA receptor agonists (Gill and Grace 2014), have attracted much attention. Our *Gad1* homozygous and heterozygous rats (combined with NMDA receptor antagonist treatment) may be a useful experimental model for the development of similar drugs. In the next phase of our research, we aim to test whether the proposed novel drugs, such as sodium benzoate, have therapeutic effects in our animal models.

## Data Availability

The raw data supporting the conclusion of this article will be made available by the authors, without undue reservation.

## References

[B1] Addington A. M. Gornick M. Duckworth J. Sporn A. Gogtay N. Bobb A. (2005). GAD1 (2q31.1), which encodes glutamic acid decarboxylase (GAD67), is associated with childhood-onset schizophrenia and cortical gray matter volume loss. Mol. Psychiatry 10, 581–588. 10.1038/sj.mp.4001599 15505639

[B2] Albani S. H. Andrawis M. M. Abella R. J. H. Fulghum J. T. Vafamand N. Dumas T. C. (2015). Behavior in the elevated plus maze is differentially affected by testing conditions in rats under and over three weeks of age. Front. Behav. Neurosci. 9, 31. 10.3389/fnbeh.2015.00031 25741257 PMC4330883

[B3] Asada H. Kawamura Y. Maruyama K. Kume H. Ding R.-G. Kanbara N. (1997). Cleft palate and decreased brain -aminobutyric acid in mice lacking the 67-kDa isoform of glutamic acid decarboxylase. Proc. Natl. Acad. Sci. 94, 6496–6499. 10.1073/pnas.94.12.6496 9177246 PMC21078

[B4] Brown J. A. Ramikie T. S. Schmidt M. J. Báldi R. Garbett K. Everheart M. G. (2015). Inhibition of parvalbumin-expressing interneurons results in complex behavioral changes. Mol. Psychiatry 20, 1499–1507. 10.1038/mp.2014.192 25623945 PMC4516717

[B5] Chang C.-H. Lin C.-H. Liu C.-Y. Chen S.-J. Lane H.-Y. (2020). Efficacy and cognitive effect of sarcosine (N-methylglycine) in patients with schizophrenia: a systematic review and meta-analysis of double-blind randomised controlled trials. J. Psychopharmacol. 34, 495–505. 10.1177/0269881120908016 32122256

[B6] Cong L. Ran F. A. Cox D. Lin S. Barretto R. Habib N. (2013). Multiplex genome engineering using CRISPR/Cas systems. Science 339, 819–823. 10.1126/science.1231143 23287718 PMC3795411

[B7] Curley A. A. Arion D. Volk D. W. Asafu-adjei J. K. Sampson A. R. Fish K. N. (2011). Cortical deficits of glutamic acid decarboxylase 67 expression in schizophrenia: clinical, protein, and cell type-specific features. Ajp 168, 921–929. 10.1176/appi.ajp.2011.11010052 PMC3273780 21632647

[B8] Fujihara K. Miwa H. Kakizaki T. Kaneko R. Mikuni M. Tanahira C. (2015a). Glutamate decarboxylase 67 deficiency in a subset of GABAergic neurons induces schizophrenia-related phenotypes. Neuropsychopharmacology 40, 2475–2486. 10.1038/npp.2015.117 25904362 PMC4538341

[B9] Fujihara K. Narita K. Suzuki Y. Takei Y. Suda M. Tagawa M. (2015b). Relationship of γ-aminobutyric acid and glutamate+glutamine concentrations in the perigenual anterior cingulate cortex with performance of Cambridge gambling task. Neuroimage 109, 102–108. 10.1016/j.neuroimage.2015.01.014 25583607

[B10] Fujihara K. Sato T. Miyasaka Y. Mashimo T. Yanagawa Y. (2020b). Genetic deletion of the 67‐kDa isoform of glutamate decarboxylase alters conditioned fear behavior in rats. FEBS Open Bio 11, 340. 10.1002/2211-5463.13065 PMC7876494 33325157

[B11] Fujihara K. Yamada K. Ichitani Y. Kakizaki T. Jiang W. Miyata S. (2020a). CRISPR/Cas9-engineered Gad1 elimination in rats leads to complex behavioral changes: implications for schizophrenia. Transl. Psychiatry. 10, 426. 10.1038/s41398-020-01108-6 33293518 PMC7723991

[B12] Gill K. Grace A. (2014). The role of α5 GABAA receptor agonists in the treatment of cognitive deficits in schizophrenia. Curr. Pharm. Des. 20, 5069–5076. 10.2174/1381612819666131216114612 24345268 PMC4074253

[B13] Guidotti A. Auta J. Davis J. M. Gerevini V. D. Dwivedi Y. Grayson D. R. (2000). Decrease in reelin and glutamic acid Decarboxylase67 (GAD67) expression in schizophrenia and bipolar disorder. Arch. Gen. Psychiatry 57, 1061–1069. 10.1001/archpsyc.57.11.1061 11074872

[B14] Hashimoto T. Arion D. Unger T. Maldonado-Avilés J. G. Morris H. M. Volk D. W. (2008). Alterations in GABA-related transcriptome in the dorsolateral prefrontal cortex of subjects with schizophrenia. Mol. Psychiatry 13, 147–161. 10.1038/sj.mp.4002011 17471287 PMC2882638

[B15] Hashimoto T. Volk D. W. Eggan S. M. Mirnics K. Pierri J. N. Sun Z. (2003). gene expression deficits in a subclass of GABA neurons in the prefrontal cortex of subjects with schizophrenia. J. Neurosci. 23, 6315–6326. 10.1523/jneurosci.23-15-06315.2003 12867516 PMC6740534

[B16] Heldt S. A. Mou L. Ressler K. J. (2012). *In vivo* knockdown of GAD67 in the amygdala disrupts fear extinction and the anxiolytic-like effect of diazepam in mice. Transl. Psychiatry 2, e181. 10.1038/tp.2012.101 23149445 PMC3565763

[B17] Kawabe K. (2017). Effects of chronic forced-swim stress on behavioral properties in rats with neonatal repeated MK-801 treatment. Pharmacol. Biochem. Behav. 159, 48–54. 10.1016/j.pbb.2017.06.009 28647564

[B18] Kawabe K. Iwasaki T. Ichitani Y. (2007). Repeated treatment with N-methyl-d-aspartate antagonists in neonatal, but not adult, rats causes long-term deficits of radial-arm maze learning. Brain Res. 1169, 77–86. 10.1016/j.brainres.2007.06.062 17706184

[B19] Keefe R. S. E. Harvey P. D. (2012). Cognitive impairment in schizophrenia. Handbook Exp. Pharmacol. 213, 11–37. 10.1007/978-3-642-25758-210.1007/978-3-642-25758-2_2 23027411

[B20] Krystal J. H. Karper L. P. Seibyl J. P. Freeman G. K. Delaney R. Bremner J. D. (1994). Subanesthetic effects of the noncompetitive NMDA antagonist, ketamine, in humans. Arch. Gen. Psychiatry 51, 199–214. 10.1001/archpsyc.1994.03950030035004 8122957

[B21] Lane H.-Y. Lin C.-H. Green M. F. Hellemann G. Huang C.-C. Chen P.-W. (2013). Add-on treatment of benzoate for schizophrenia. JAMA Psychiatry 70, 1267–1275. 10.1001/jamapsychiatry.2013.2159 24089054

[B22] Lee G. Zhou Y. (2019). NMDAR hypofunction animal models of schizophrenia. Front. Mol. Neurosci. 12, 185. 10.3389/fnmol.2019.00185 31417356 PMC6685005

[B23] Leo D. Sukhanov I. Zoratto F. Illiano P. Caffino L. Sanna F. (2018). Pronounced hyperactivity, cognitive dysfunctions, and BDNF dysregulation in dopamine transporter knock-out rats. J. Neurosci. 38, 1959–1972. 10.1523/JNEUROSCI.1931-17.2018 29348190 PMC5824739

[B24] Lewis D. A. Sweet R. A. (2009). Schizophrenia from a neural circuitry perspective: advancing toward rational pharmacological therapies. J. Clin. Invest. 119, 706–716. 10.1172/JCI37335 19339762 PMC2662560

[B25] Li J.-T. Su Y.-A. Wang H.-L. Zhao Y.-Y. Liao X.-M. Wang X.-D. (2016). Repeated blockade of NMDA receptors during adolescence impairs reversal learning and disrupts GABAergic interneurons in rat medial prefrontal cortex. Front. Mol. Neurosci. 9, 17. 10.3389/fnmol.2016.00017 26973457 PMC4776083

[B26] Lin C.-H. Lin C.-H. Chang Y.-C. Huang Y.-J. Chen P.-W. Yang H.-T. (2018). Sodium benzoate, a D-amino acid oxidase inhibitor, added to clozapine for the treatment of schizophrenia: a randomized, double-blind, placebo-controlled trial. Biol. Psychiatry 84, 422–432. 10.1016/j.biopsych.2017.12.006 29397899

[B43] Lin C.-Y. Liang S.-Y. Chang Y.-C. Ting S.-Y. Kao C.-L. Wu Y.-H. (2017). Adjunctive sarcosine plus benzoate improved cognitive function in chronic schizophrenia patients with constant clinical symptoms: A randomised, double-blind, placebo-controlled trial. World J. Biol. Psychiatry 18, 357–368. 10.3109/15622975.2015.1117654 26691576

[B27] Ma Y.-N. Sun Y.-X. Wang T. Wang H. Zhang Y. Su Y.-A. (2020). Subchronic MK-801 treatment during adolescence induces long-term, not permanent, excitatory-inhibitory imbalance in the rat hippocampus. Eur. J. Pharmacol. 867, 172807. 10.1016/j.ejphar.2019.172807 31751575

[B28] Miyata S. Kumagaya R. Kakizaki T. Fujihara K. Wakamatsu K. Yanagawa Y. (2019). Loss of glutamate decarboxylase 67 in somatostatin-expressing neurons leads to anxiety-like behavior and alteration in the akt/gsk3β signaling pathway. Front. Behav. Neurosci. 13, 131. 10.3389/fnbeh.2019.00131 31275123 PMC6591520

[B29] Noda Y. Yamada K. Furukawa H. Nabeshima T. (1995). Enhancement of immobility in a forced swimming test by subacute or repeated treatment with phencyclidine: a new model of schizophrenia. Br. J. Pharmacol. 116, 2531–2537. 10.1111/j.1476-5381.1995.tb15106.x 8581295 PMC1909055

[B30] Nullmeier S. Elmers C. D’Hanis W. Sandhu K. V. K. Stork O. Yanagawa Y. (2020). Glutamic acid decarboxylase 67 haplodeficiency in mice: consequences of postweaning social isolation on behavior and changes in brain neurochemical systems. Brain Struct. Funct. 225, 1719–1742. 10.1007/s00429-020-02087-6 32514634 PMC7321906

[B31] Obata K. (2013). Synaptic inhibition and ^|^gamma;-aminobutyric acid in the mammalian central nervous system. Proc. Jpn. Acad. Ser. B 89, 139–156. 10.2183/pjab.89.139 23574805 PMC3669732

[B32] Pérez M. Á. Morales C. Santander O. García F. Gómez I. Peñaloza-Sancho V. (2019). Ketamine-treatment during late adolescence impairs inhibitory synaptic transmission in the prefrontal cortex and working memory in adult rats. Front. Cell. Neurosci. 13, 372. 10.3389/fncel.2019.00372 31481877 PMC6710447

[B33] R Core Team (2017). A language and environment for statistical computing. Available at: http://www.r-project.org. (Accessed December 15, 2020)

[B44] Remington G. Foussias G. Fervaha G. Agid O. Takeuchi H. Lee J. (2016). Treating negative symptoms in schizophrenia: an update. Curr. Treat. Options Psychiatry 3, 133–150. 10.1007/s40501-016-0075-8 27376016 PMC4908169

[B34] Rodgers R. J. Dalvi A. (1997). Anxiety, defence and the elevated plus-maze. Neurosci. Biobehav. Rev. 21, 801–810. 10.1016/s0149-7634(96)00058-9 9415905

[B35] Rujescu D. Bender A. Keck M. Hartmann A. M. Ohl F. Raeder H. (2006). A pharmacological model for psychosis based on N-methyl-D-aspartate receptor hypofunction: molecular, cellular, functional and behavioral abnormalities. Biol. Psychiatry 59, 721–729. 10.1016/j.biopsych.2005.08.029 16427029

[B36] Silveri M. M. Sneider J. T. Crowley D. J. Covell M. J. Acharya D. Rosso I. M. (2013). Frontal lobe γ-aminobutyric acid levels during adolescence: associations with impulsivity and response inhibition. Biol. Psychiatry 74, 296–304. 10.1016/j.biopsych.2013.01.033 23498139 PMC3695052

[B37] Uchida T. Furukawa T. Iwata S. Yanagawa Y. Fukuda A. (2014). Selective loss of parvalbumin-positive GABAergic interneurons in the cerebral cortex of maternally stressed gad1-heterozygous mouse offspring. Transl. Psychiatry 4, e371. 10.1038/tp.2014.13 24618690 PMC3966041

[B38] Uchida T. Oki Y. Yanagawa Y. Fukuda A. (2011). A heterozygous deletion in the glutamate decarboxylase 67 gene enhances maternal and fetal stress vulnerability. Neurosci. Res. 69, 276–282. 10.1016/j.neures.2010.12.010 21185888

[B39] Umemori J. Takao K. Koshimizu H. Hattori S. Furuse T. Wakana S. (2013). ENU-mutagenesis mice with a non-synonymous mutation in grin1 exhibit abnormal anxiety-like behaviors, impaired fear memory, and decreased acoustic startle response. BMC Res. Notes 6, 203. 10.1186/1756-0500-6-203 23688147 PMC3674941

[B40] Unal G. Ates A. Aricioglu F. (2018). Agmatine-attenuated cognitive and social deficits in subchronic MK-801 model of schizophrenia in rats. Psychiatry Clin. Psychopharmacol. 28, 245–253. 10.1080/24750573.2018.1426696

[B41] Uttl L. Petrasek T. Sengul H. Svojanovska M. Lobellova V. Vales K. (2018). Chronic MK-801 application in adolescence and early adulthood: a spatial working memory deficit in adult long-Evans rats but no changes in the hippocampal NMDA receptor subunits. Front. Pharmacol. 9, 42. 10.3389/fphar.2018.00042 29487522 PMC5816576

[B42] Volk D. W. Austin M. C. Pierri J. N. Sampson A. R. Lewis D. A. (2000). Decreased glutamic acid Decarboxylase67 messenger RNA expression in a subset of prefrontal cortical γ-aminobutyric acid neurons in subjects with schizophrenia. Arch. Gen. Psychiatry 57, 237–245. 10.1001/archpsyc.57.3.237 10711910

