# Supplementary material for: Behavioral Consequences of a Combination of Gad1 Haplodeficiency and Adolescent Exposure to an NMDA Receptor Antagonist in Long-Evans Rats
Source: Front Pharmacol. 2021 Mar 30;12:646088. doi: 10.3389/fphar.2021.646088 (PMC8042137; doi:10.3389/fphar.2021.646088)
Supplement: Supplementary file 2 [file datasheet2.pdf]

Supplementary Material

Fujihara et al.

Behavioral consequences of a combination of *Gad1* haplodeficiency and adolescent exposure to an NMDA receptor antagonist in Long-Evans rats

1 Supplementary Methods

1.1 Animals

The rooms for breeding and experiments were maintained at 22 ± 3 °C with a 12-h light-dark cycle (lights on at 6:00, lights off at 18:00). The rats were group-housed (six rats per cage). Genotyping polymerase chain reactions was carried out using the following primers: 5-ACTGGGCCATTGTTCCAGCTCCA-3 (primer 1), 5-GCTCTCTCACGAGTATGCCCTTGCT-3 (primer 2), and 5-CGAGCTGGAGAAGGGGGAAGAAGAT-3 (primer 3). See our previous report for details (Fujihara et al., 2020).

1.2 Acoustic startle response and prepulse inhibition test

The acoustic startle test and prepulse inhibition test were carried out as described previously with minor modifications (Fujihara et al., 2015, 2020). The acoustic startle response was evaluated with three different intensities (100, 110, and 120 dB). The white noise stimuli (40 ms) of each intensity were presented in a quasi-random order and at random intertrial intervals (10–20 s). In the prepulse

inhibition (PPI) session, rats experienced five different types of stimuli: no stimulus; startle stimulus (120 dB, 40 ms) only; prepulse 70 dB (20 ms, lead time 100 ms) and pulse 120 dB; prepulse 75 dB (20 ms, lead time 100 ms) and pulse 120 dB; and prepulse 80 dB (20 ms, lead time 100 ms) and pulse 120 dB. Each trial was repeated 10 times in a quasi-random order with random intertrial intervals (10–20 s). PPI was defined as the percent decline in the startle response: 100 − [(startle amplitude after prepulse and pulse)/(startle amplitude after pulse only)] × 100.

1.2 Y-maze test

The Y-maze test was used to screen for impairment of spatial working memory without food restriction stress (Fujihara et al., 2020). The apparatus comprised three identical arms separated by 120 degrees from each other. The length of each arm was 60 cm, and its width was 6 cm (O’Hara, Tokyo, Japan). The center of the maze was illuminated at 50 lx. Each rat was placed at the end of one arm and allowed to move freely through the maze for 8 min. Spontaneous alternation behaviors were counted with TimeYM software (O’Hara & Co., Ltd., Tokyo, Japan).

1.3 Elevated plus-maze test

The elevated plus-maze test was performed as described previously (Fujihara et al., 2020, 2021). The maze consisted of two open arms and two closed arms (O’Hara, Tokyo, Japan). Each arm was of the same size (10 cm × 50 cm), and the closed arm had 35-cm-high transparent walls. The apparatus was placed 50 cm above the floor. The brightness of the center area was 50 lx. Each rat was allowed to

move freely in the maze for 10 min. The durations spent in the closed arms, open arms, and center area were measured with TimeEP software (O'Hara & Co., Ltd., Tokyo, Japan).

2.4 Open field test

A 90-cm × 90-cm open field apparatus (O'Hara, Tokyo, Japan) was used as described previously (Fujihara et al., 2020, 2021). The center of the arena was illuminated at 50 lx. Each rat was allowed to move freely during a 5-min session (Miyata et al., 2019). The behavior of the rats was recorded with an attached CCD camera. The distance traveled during a session and the time spent in the central area (36% of the field) was assessed accordingly. Furthermore, we measured the rearing behaviors automatically with an infrared beam sensor. Data analysis was carried out using the automated software TimeOFCR1 (O'Hara & Co., Ltd., Tokyo, Japan).

1.5 Social interaction test

The social behaviors of the rats were tested as described previously (Fujihara et al., 2020). An open field apparatus was used for this test. Two identical wire cages were placed inside the arena diagonally. First, rats were allowed to move freely around the arena for 10 min for habituation. A stranger rat younger than the subjects was enclosed in one of the wire cages immediately after habituation. Then, the rat to be tested was put back in the arena (sociability test). The amount of time the rats spent around the cages was measured for 5 min. After a 5-min interval, we placed a different stranger rat in the empty wire cage, and again, the time spent around each cage was recorded for 5 min (social novelty preference test). The data were analyzed using TimeSSI (O'Hara & Co., Ltd., Tokyo, Japan).

1.6 Porsolt forced Swim test

The forced swim test was carried out according to a previously described protocol with minor modifications (Fujihara et al., 2020). Plexiglas cylinders (20 cm diameter × 45 cm height; O’Hara, Tokyo, Japan) were filled with water (25 cm depth), whose temperature was maintained at 22 ± 2°C. On the first day, each rat was placed in water for 10 min. On the second day, each rat was put into water again, and its behavior was recorded for 6 min by a CCD camera. The data were analyzed using TimeFZ1 (O’Hara & Co., Ltd., Tokyo, Japan).

1.7 Cued fear conditioning test

The cued fear conditioning test was conducted in a box surrounded by a sound-attenuated chamber (O'Hara & Co., Ltd., Tokyo, Japan) as described previously (Fujihara et al., 2021). This test consisted of a conditioning trial (day 1) and 4-day cued test trials (day 2 to day 5) (Supplementary Figure S3). The rats were placed in a clear acrylic conditioning chamber (33 cm × 25 cm × 28 cm) equipped with a stainless-steel grid floor connected to a shock generator. The brightness of the chamber was set at 120 lx. On day 1, the rats were habituated to the conditioning chamber for 180 s. Immediately after, they received five auditory tones (CS; 10 kHz, 65 dB, duration: 20 s) with an 80 s interval; each tone was delivered simultaneously with a foot shock at the end of it (US; 0.7 mA, duration: 1 s). One hundred seconds after the last foot shock, the rats were returned to their home cages. A cued test was performed in a chamber with different contexts. The test chamber differed from the conditioning chamber in brightness (30 lx), color (black), and shape (triangular prism, 33 cm on one side and 31.5 cm high). The schedule for the cue test was the same as that for the conditioning, except that no foot

shock was administered in this case. The acquired cued fear was tested through five CS periods (without foot shock). From day 3 to day 5, we repeated the same cued test to assess extinction learning. The percentage of time that the rats exhibited freezing responses was measured as an index of fear memory. The decrease in freezing level in each rat during the fear extinction test was expressed as a ratio to the freezing level of day 2 (extinction rate [%]). The data were analyzed using TimeFZ1 (O’Hara & Co., Ltd., Tokyo, Japan). The freezing time was automatically determined by the software.

2 Supplementary Talbe

Behavioral test	Age
Injection of MK-801 or saline	4–6 weeks (P30－43)
Acoustic startle response and prepulse inhibition test	7 weeks
Y-maze test	8 weeks
Elevated plus-maze test	9 weeks
Open field test	10–12 weeks
Social interaction test	15–16 weeks
Porsolt forced swim test	19 weeks
Cued fear conditioning test	20–23 weeks

Supplementary Table 1. Schedule of the behavioral experiment.

3 Supplementary Figures

3.1 Supplementary Figure 1

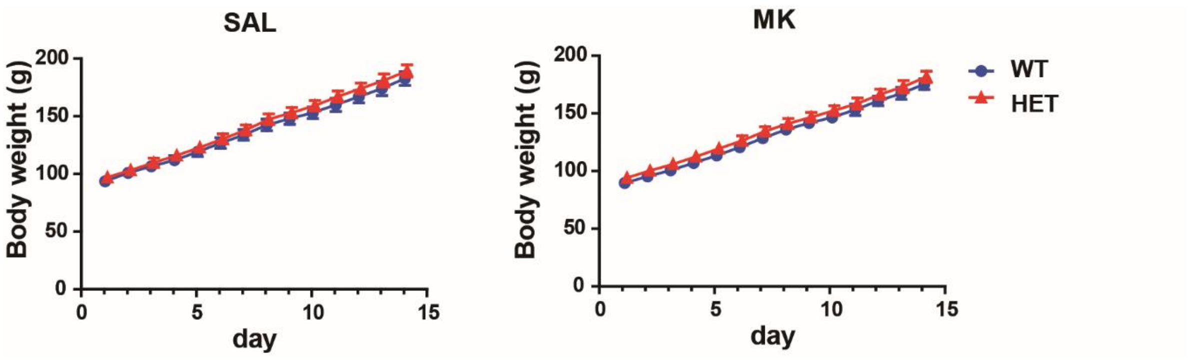

Supplementary Figure 1. The time course of body weight changes during MK-801 administration. Saline or MK-801 (0.2 mg/kg) was injected intraperitoneally from P30 to P43 for 14 days. MK-801 did not affect the growth curve in each genotype (genotype, $F(1,36) = 1.604$, $p = 0.213$, $\eta_p^2 = 0.043$; drug, $F(1,36) = 2.039$, $p = 0.162$, $\eta_p^2 = 0.054$; genotype $\times$ drug, $F(1,36) = 0.005$, $p = 0.943$, $\eta_p^2 = 0.000$). The results are presented as average $\pm$ SEM. Data were analyzed using a three-way repeated-measures ANOVA. WT: wild-type, HET: *Gad1*$^{+/-}$, SAL: saline, MK: MK-801.

3.2 Supplementary Figure 2

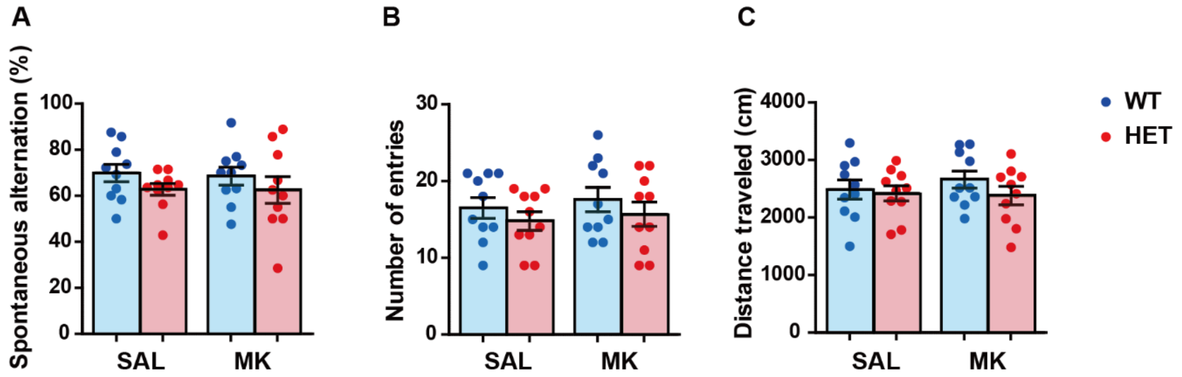

Supplementary Figure 2. The result of the Y-maze test. (A) The spontaneous alternation rate (genotype, $F(1,36) = 2.440$, $p = 0.127$, $\eta_p^2 = 0.064$; drug, $F(1,36) = 0.038$, $p = 0.847$, $\eta_p^2 = 0.001$; genotype × drug, $F(1,36) = 0.017$, $p = 0.898$, $\eta_p^2 = 0.000$), (B) number of entries to the arms (genotype, $F(1,36) = 1.554$, $p = 0.221$, $\eta_p^2 = 0.041$; drug, $F(1,36) = 0.480$, $p = 0.493$, $\eta_p^2 = 0.013$; genotype × drug, $F(1,36) = 0.005$, $p = 0.945$, $\eta_p^2 = 0.000$) and (C) distance traveled (genotype, $F(1,36) = 1.278$, $p = 0.266$, $\eta_p^2 = 0.034$; drug, $F(1,36) = 0.200$, $p = 0.657$, $\eta_p^2 = 0.006$; genotype × drug, $F(1,36) = 0.496$, $p = 0.486$, $\eta_p^2 = 0.014$) were comparable between the four groups. The results are presented as average ± SEM. Data were analyzed using a two-way ANOVA. WT: wild-type, HET: *Gad1*$^{+/-}$, SAL: saline, MK: MK-801.

3.3 Supplementary Figure 3

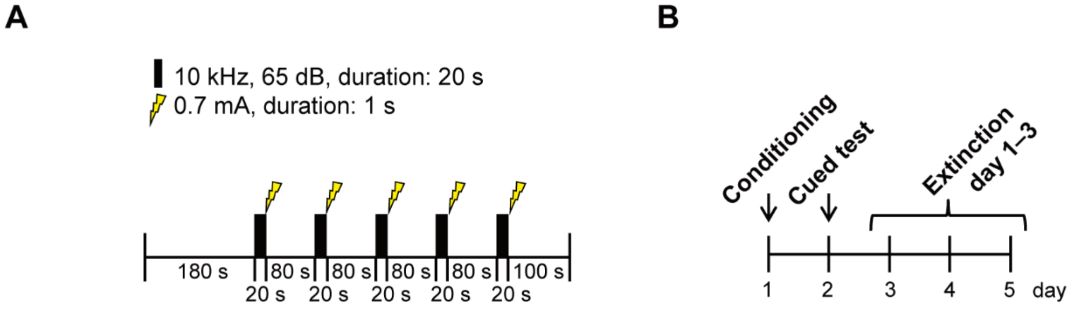

Supplementary Figure 3. A schematic diagram of the cued fear conditioning test. (A) On the first day, each rat was trained in a conditioning chamber. After 180-s habituation, five tone-shock pairings were administered. At the end of the 20-s tone, a foot shock (0.7 mA, 1 s) was delivered. The interval between each tone was 80 s. (B) The schedule of the fear conditioning and extinction. On the first day of the experiment, we performed the training in (A). On the second day, we performed a cued test in a chamber with a different context. The same tones as the first day were delivered. The cued test was repeated to assess fear extinction for additional three days.
